# The Dual Role of B Cells in the Tumor Microenvironment: Implications for Cancer Immunology and Therapy

**DOI:** 10.3390/ijms252111825

**Published:** 2024-11-04

**Authors:** Hao Yang, Zhiru Zhang, Jijun Li, Kun Wang, Wanting Zhu, Yingyue Zeng

**Affiliations:** School of Life Science, Liaoning University, Shenyang 110036, China; yanghao0128in@163.com (H.Y.); zhangzhiru281220@163.com (Z.Z.); 15897151688@163.com (J.L.); 18834922568@163.com (K.W.); 13894462625@163.com (W.Z.)

**Keywords:** tumor microenvironment, B cell, anti-tumor therapy, B cell heterogeneity

## Abstract

The tumor microenvironment (TME) is a complex and heterogeneous tissue composed of various cell types, including tumor cells, stromal cells, and immune cells, as well as non-cellular elements. Given their pivotal role in humoral immunity, B cells have emerged as promising targets for anti-tumor therapies. The dual nature of B cells, exhibiting both tumor-suppressive and tumor-promoting functions, has garnered significant attention. Understanding the distinct effects of various B cell subsets on different tumors could pave the way for novel targeted tumor therapies. This review provides a comprehensive overview of the heterogeneous B cell subsets and their multifaceted roles in tumorigenesis, as well as the therapeutic potential of targeting B cells in cancer treatment. To develop more effective cancer immunotherapies, it is essential to decipher the heterogeneity of B cells and their roles in shaping the TME.

## 1. Introduction

The tumor microenvironment (TME) has emerged as a critical determinant of cancer progression, with increasing recognition of its multifaceted influence on tumor biology. The TME is a complex ecosystem composed of various cellular and non-cellular components, including tumor cells, stromal cells, and immune cells. This microenvironment is often likened to a symbiotic relationship between “soil” and “seed,” as it co-drives tumor growth, invasion, and metastasis [1,2,3]. Emerging evidence underscores the critical role of immune cells in shaping the TME and driving tumorigenesis. Within the TME, lymphoid aggregates of varying size and complexity are often observed, typically composed of T and B cells. These structures, collectively known as tertiary lymphoid structures (TLSs), can range from small, disorganized clusters to larger, more organized formations [4]. B cells, a major constituent of the TME, especially within TLSs, have been extensively studied for their dual roles in both tumor suppression and promotion. Because of their ability to produce antibodies and enhance T cell responses, B cells are not mere passive bystanders but active drivers of tumor progression and treatment outcomes [5,6]. Pro-tumor regulatory B cells (Bregs) often secrete immunosuppressive cytokines, thereby driving immune suppression and tumor progression [7,8,9]. Conversely, anti-tumor B cells can inhibit tumor development by producing tumor-specific antibodies [10]. This review delves into the diversity of B cells within tumors, their dual roles in promoting and inhibiting tumor growth, and current therapeutic strategies targeting B cells in cancer.

### B Cell Life Cycle: From Origin to Function

**Figure 1:**
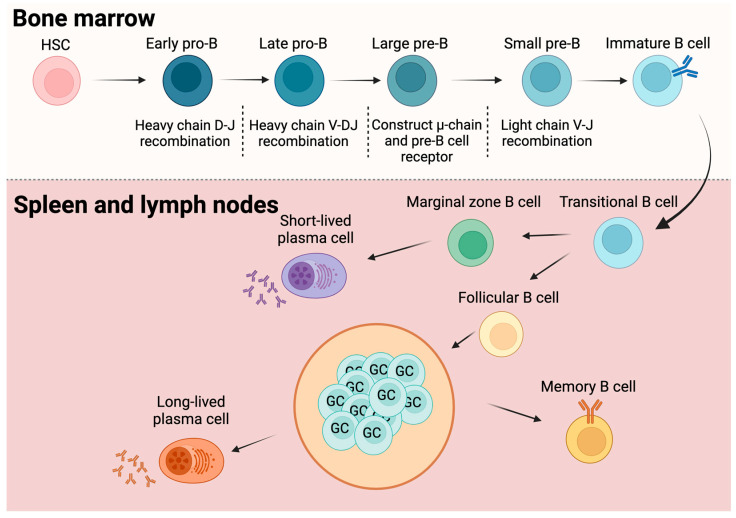
B cell life cycle: from origin to function. B cell development begins with hematopoietic stem cells (HSCs) differentiating into CLPs, followed by pro-B cell stages. During the early pro-B cell stage, heavy-chain DJ gene segments undergo recombination. Subsequently, the upstream V region joins the rearranged DJ segment in the late pro-B cell stage. Successful completion of this process forms a full μ heavy chain, leading to the pre-B cell stage. The recombination of the light-chain VJ gene segment marks the transition to the small pre-B cell phase, ultimately resulting in cells expressing surface IgM but not yet fully mature. Immature B cells enter the spleen or lymph nodes as transitional B cells and differentiate into two distinct lineages. Marginal zone B cells can rapidly differentiate into short-lived plasma cells upon antigen encounter, while follicular B cells form germinal centers (GCs) with T cell help and eventually differentiate into long-lived plasma cells and memory B cells. Created in BioRender. (accessed on 10 October 2024) https://BioRender.com/k00z299.

The development of B cells starts with undifferentiated hematopoietic stem cells (HSCs), which undergo a complex maturation process within the bone marrow (Figure 1). Early in their development within the bone marrow, B cells undergo immunoglobulin gene rearrangement to acquire functional antigen receptors [11]. B cell development begins with HSCs differentiating into common lymphoid progenitors (CLPs), followed by pro-B cell stages. During the early pro-B cell stage, heavy-chain DJ gene segments undergo recombination. Subsequently, the upstream V region joins the rearranged DJ segment in the late pro-B cell stage. The successful completion of this process forms a full μ heavy chain, leading to the pre-B cell stage. The recombination of the light-chain VJ gene segment marks the transition to the small pre-B cell phase, ultimately resulting in cells expressing surface immunoglobulin M (IgM) but not yet fully mature [12,13,14]. To maintain self-tolerance, B cells undergo negative selection at this stage, eliminating self-reactive cells through mechanisms such as apoptosis, receptor editing, and clonal deletion [15,16,17].

Having become independent of stromal cell support, immature B cells migrate to the spleen and differentiate into transitional B cells (Figure 1). Transitional B cells in the spleen differentiate into two populations: marginal zone B cells, which respond to T cell-independent antigens and rapidly become short-lived plasma cells secreting IgM, and follicular B cells [12,18]. Follicular B cells, in conjunction with helper T cells (Th), are activated by Th-derived cytokines, entering germinal centers (GCs). These GCs can be found in both the spleen and lymph nodes [19]. Follicular B cells differentiate into memory B cells or long-lived plasma cells, which then produce high-affinity antibodies after undergoing a series of mutations, selections, and proliferations [20]. Memory B cells and plasma cells, located in the periphery or bone marrow, are quickly reactivated upon antigen re-exposure, launching a secondary immune response that rapidly and effectively generates antibodies, safeguarding the body.

## 2. Heterogeneity of B Cells in the TME

Historically, tumor immunology has primarily focused on the T cell-mediated immune response against cancer. In contrast, the role of B cells within the TME has been relatively under-investigated [21]. Recent research highlights the importance of B cells within the TME, due to their humoral immune capabilities, suggesting a potential protective role against tumor progression [22].

B cells, a pluripotent lineage, exhibit a multifaceted functionality, serving as antibody-secreting cells, immunoregulatory cells, and antigen-presenting cells (APCs) [23,24,25]. The versatile nature of tumor-infiltrating B cells (TIL-Bs) manifests itself in their ability to toggle between acting as guardians against malignancy and unwitting accomplices in tumor expansion. The precise mechanisms governing the specificity and function of B cells, especially in the context of cancer, remain largely unexplored, despite their dual potential.

This uncertainty highlights the complex relationship between B cell subsets and tumor development. Recent studies have demonstrated that the quantity and developmental stage of B cells differ significantly across various cancers, leading to the identification of 15 distinct B cell subtypes and 10 plasma cell subtypes [26]. This diversity emphasizes the significance of thoroughly understanding B cell heterogeneity within the TME to fully evaluate its clinical implications for patient populations. A deeper understanding of B cell heterogeneity is essential for developing targeted therapeutic strategies that fully leverage the potential of B cells in cancer immunotherapy.

### 2.1. B Cell Spectrum: The Varied Faces of Plasma Cells in Oncology

B cell subsets display a variety of functions, one of which is antibody secretion. Through the production of high-affinity antibodies, B cells, particularly plasma cells, contribute to the body’s anti-tumor response by initiating humoral immunity. Plasma cell infiltration has been documented in multiple cancers, such as prostate cancer, breast cancer, ovarian cancer, melanoma, and head and neck squamous cell carcinoma [27,28,29,30,31]. There is considerable variability in the immunoglobulin phenotype transitions of these cells among different tumor types and individuals. In hepatocellular carcinoma, plasma cells predominantly exhibit high expression of germline γ-chain constant regions (Cγ), indicative of IgG class switch recombination [32]. The class switch recombination pattern in human breast cancer is complex, with both IgG and IgA isotypes being detected [33]. In prostate cancer models, on the other hand, IgA class switch recombination is detected [27]. IgG+ plasma cell infiltration is positively correlated with the magnitude of Th1/Th17 immune responses in inflamed tumor tissues [34]. IgA+ plasma cells, which frequently exhibit an immunosuppressive phenotype marked by the expression of IL-10 and PD-L1, are associated with diminished therapeutic efficacy [27]. Consistently, IgG+ plasma cell precursors preferentially express chemokine receptors CXCR3 and CCR4 associated with Th1 responses [35], whereas IgA+ plasma cell precursors preferentially express homing receptors CCR10 and CCR9 [36]. IgA+ plasma cells express homing receptors that can be recruited by CCL28, which also recruits immunosuppressive T cells, further associating IgA+ plasma cells with immunosuppression, unlike IgG+ plasma cells [37]. The functional profile of B cells in the TME is notably different from that observed in other disease contexts. Atypical memory B cells in tumors display an elevated expression profile of stress- and activation-associated genes (such as HSPA1A, NR4A1, and CD69), whereas in autoimmune diseases, these cells exhibit a more naïve gene expression pattern, as evidenced by the expression of IGHD. The diversity of B cell functions is underpinned by the selective expression of these genes [26]. These findings highlight the intricate interplay between different B cell subtypes and tumors, suggesting their distinct roles in tumor progression.

The high specificity of tumor-infiltrating plasma cells for tumor-associated antigens (TAAs) makes them crucial players in anti-tumor immunity. It has been demonstrated that tumor-infiltrating plasma cells generate antibodies that specifically target TAAs in various cancers [38,39,40]. However, effective tumor-specific B cell receptor (BCR) clone expansions are far fewer than the potential neoantigen numbers in most tumor types, indicating impaired local humoral immunity [41]. Tumors with dominant specific antigens, like HER2+ breast cancer and MART-1+ melanoma, show a correlation between plasma cell infiltration and improved therapeutic outcomes [42,43,44]. In contrast, in tumors without identified or dominant antigens, humoral immunity’s tumor-killing effect is lower [45]. The plasma cell component in tumors is complex, with some virus-specific plasma cells effectively combating tumors, while others specific to self-antigens promote inflammation, driving tumor progression [46]. Consequently, the highly heterogeneous subgroups of plasma cells form a complex TME, determining the strength and direction of humoral immunity and tumor progression under conditions of viral background, immunogenicity, and the immune contents of tumors.

### 2.2. B Cell Spectrum: Antigen Presenters and Immune Modulators

Beyond antibody-secreting B cells in the TME, other B cell populations can be summarized as antigen presenters and immune modulators, with dendritic cells (DCs) serving as prime examples of potent antigen presentation capabilities. DCs, initially characterized over 50 years ago, have been established as the most potent APCs [47]. APCs present antigens to T cells through a three-stage process: antigen capture, processing, and presentation on the major histocompatibility complex (MHC). B cells, acting as professional antigen-presenting cells, can activate CD4+ T cells via the classical MHC class II pathway and can also cross-present antigens to CD8+ T cells [48]. B cells, although capable of functioning as APCs, are often overlooked, possibly due to their limited non-specific antigen capture and their infrequent presence in tissues [49]. However, B cells exhibit a stronger binding capacity to antigens, demonstrating a higher affinity for antigens compared to dendritic cells [50]. In non-small cell lung cancer (NSCLC), activated TIL-B cells correlate with effector T cell responses, whereas exhausted TIL-B cells are linked to regulatory T cell phenotypes [51]. A study in 2021 encompassing ten types of cancer identified an antigen-presenting B cell subset defined by high CD86 expression and downregulated CD21. This subset was increased in nine TMEs with site specificity, except for renal cell carcinoma, and could induce specific T cell responses [52]. Research on antigen-presenting B cells in the TME is relatively sparse. A deeper understanding of the mechanisms underlying the function of antigen-presenting B cell subsets in the TME is crucial for harnessing their antigen-presenting capabilities to augment anti-tumor immune responses. This line of research could potentially open new avenues for enhancing the efficacy of cancer immunotherapies by targeting B cell-mediated antigen presentation.

Within the TME, a subset known as regulatory B cells (Bregs) exists, mirroring the function of regulatory T cells (Tregs). Bregs act as critical immune modulators by tempering the immune response to maintain balance [53]. Bregs are considered a significant source of tumor-promoting activities, capable of dampening T cell immune responses within the tumor. Initially, this subset was characterized by IL-10 expression and a phenotype of CD1highCD5+ in murine peripheral blood [54]. CD5+ regulatory B cells are present in murine bladder cancer, while in prostate cancer, CD138+IgA+PD-L1+ B cells promote tumor progression by secreting the immunosuppressive cytokine IL-10 [27,55]. Murine tumors harbor IL-10-producing B cells analogous to those found in humans, such as the PD-1high B cell subsets identified in human liver cancer [56]. In contrast to the minimal infiltration of PD-1high B cell subsets observed in murine liver cancer, human liver cancer exhibits significant B cell infiltration, suggesting distinct mechanisms underlying B cell recruitment in these species. Beyond IL-10-expressing immunosuppressive B cells, in liver cancer, an FcγRIIlow/- Breg subset has been discovered, functioning as an inhibitory receptor to suppress the production of inflammatory cytokines in B cells [57]. Furthermore, a variety of Breg types have been identified in primary or metastatic tumors, including TNF-α+, STAT3+Bregs, and others [58,59]. The heterogeneity of Bregs reflects the intricate network of immune cell interactions shaping the TME. Gaining a comprehensive understanding of the heterogeneity of tumor B cells is crucial for elucidating the mechanisms underlying tumor progression and for the development of targeted therapies aimed at B cell-related tumor treatments. This knowledge could lead to innovative therapeutic strategies targeting specific B cell subsets implicated in tumor regulation, potentially improving the effectiveness of cancer treatments.

## 3. The Dual Role of B Cells in the Tumor Microenvironment

**Figure 2:**
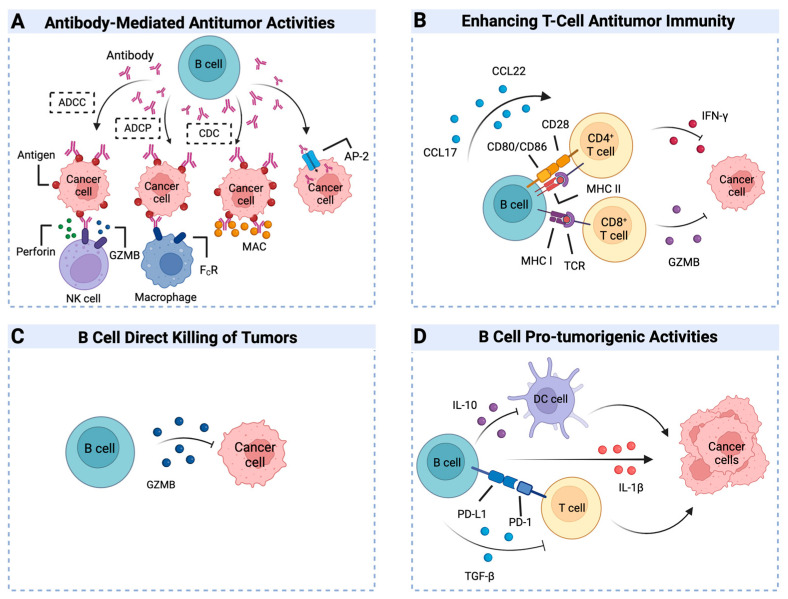
B cells play dual roles in the TME. B cells within the TME exhibit dual roles, executing anti-tumor activities through three primary mechanisms: (**A**) The antibody-mediated anti-tumor impact chiefly functions via the creation of antigen–antibody complexes, leveraging three crucial processes—ADCC, ADCP, and CDC—to accurately pinpoint and eradicate malignant cells. Furthermore, antibodies augment their intracellular access by engaging with adaptor proteins, exemplified by AP-2, thus enhancing their uptake into neoplastic cells and empowering potent intracellular tumor suppression. (**B**) As professional antigen-presenting cells (APCs), B cells have the capability to present antigens to T cells and activate them, thereby contributing to the inhibition of tumor progression. (**C**) Direct tumor cell killing is achieved by B cells producing granzyme B, directly targeting and destroying tumor cells. (**D**) B cells can facilitate tumor progression by secreting immunosuppressive factors and influencing the activity of T cells and DCs, creating an immunosuppressive TME that supports tumor growth and evasion of immune surveillance. Abbreviations: ADCP, antibody-dependent cell-mediated phagocytosis; ADCC, antibody-dependent cellular cytotoxicity; CDC, complement-dependent cytotoxicity; GZMB, granzyme B; NK cell, natural killer cells; FC R, Fc receptors; MAC, membrane attack complex; CCL17, chemokine ligand 17; CCL22, chemokine ligand 22; MHC, major histocompatibility complex; TCR, T cell receptor; DC cell, dendritic cells; IFN-γ, interferon-gamma; IL-10, interleukin-10; PD-L1, programmed death ligand 1; PD-1, programmed cell death protein 1; TGF-β, transforming growth factor beta; IL-1β, interleukin-1 beta. Created in BioRender. (accessed on 10 October 2024) https://BioRender.com/l23c546.

The presence of B cells and plasma cells in tumors is often linked to clinical outcomes in a variety of cancers, such as liver cancers, endometrial, breast, and ovarian [60,61,62,63]. TIL-Bs are present in diverse solid tumors, displaying a dual role in promoting or suppressing tumor growth (Figure 2). On one hand, TIL-Bs can inhibit tumor progression through various mechanisms, including immunoglobulin secretion, T cell response stimulation, and direct cancer cell elimination. The secreted immunoglobulins can curb tumor growth via mechanisms such as antibody-dependent cellular cytotoxicity (ADCC), antibody-dependent cell-mediated phagocytosis (ADCP), and complement-dependent cytotoxicity (CDC). B cells, functioning as APCs, can trigger T cell responses, resulting in T cell activation, clonal expansion, and the formation of memory T cells, ultimately limiting tumor growth. Another mechanism of anti-tumor activity involves B cells producing granzyme B (GZMB) to directly target and destroy tumor cells. Alternatively, B cells can contribute to an environment conducive to tumor growth via the release of cytokines that dampen immune surveillance. A subset of regulatory B cells, by producing IL-10 and TGF-β, promotes tumor growth and metastasis [7,64,65]. TIL-Bs, residing in the TME, interact with multiple immune cell types, including CD4+ T cells, CD8+ T cells, natural killer cells (NK), macrophages, and dendritic cells [30,60,66,67,68].

In subsequent sections, we explore studies that underscore the dualistic function of B cells within the TME, investigating their capacity that spans from curbing tumor progression to enabling its advancement. A deeper understanding of the mechanisms underlying these opposing roles of B cells is essential for designing targeted therapies that can harness their anti-tumor potential while mitigating their pro-tumor effects, potentially leading to more effective cancer treatments. We will explore the mechanisms underlying the dual nature of B cells in the context of tumor immunity.

### 3.1. Engaging B Cells in the War on Cancer

B cells contribute significantly to anti-tumor immunity by secreting immunoglobulins, and effective B cell infiltration and accumulation of antibody-producing cells within the TME are crucial for robust anti-tumor responses. Antibodies primarily mediate their effects through ADCC, ADCP, and CDC, as evidenced by increasing research (Figure 2). ADCC is triggered by the interaction of antibody–antigen complexes with Fc receptors on myeloid cells and NK cells, leading to the cytotoxic elimination of target cells. The presence of increased tumor-infiltrating plasma cells in prostate cancer is linked to enhanced NK cell activation and IgG expression, correlating with improved clinical outcomes [69]. The specific mechanism can be succinctly outlined as follows: upon the engagement of antibody–antigen complexes with Fcγ receptors on NK cells, these cells discharge perforins and granzymes. Following the pore formation facilitated by perforins, granzymes gain access into the tumor cells and ignite apoptotic signaling cascades encompassing caspase-3 and caspase-9, consequently orchestrating programmed cell demise [70,71]. Beyond this, ADAM17 modulates NK cell ADCC activity at the surface of tumor cells by cleaving Fcγ receptors. Upon the activation of Fcγ receptors, NK cell effector functions and cytotoxicity are augmented via the PI3K/Akt pathway [72]. Similarly, in a mouse model of pancreatic cancer, serum antibodies were found to induce ADCC [73]. Likewise, the presence of IgG in ovarian cancer and renal cell carcinoma is linked to myeloid cell infiltration and NK cell-mediated ADCC [74,75]. ADCP is initiated when antibody–antigen complexes bind to Fc receptors on myeloid cells, triggering phagocytosis of target cells. The principal signaling pathways involved in ADCP-mediated tumor killing encompass those activated following the engagement of antibody–antigen complexes with Fcγ receptors on macrophages. Prominent among these are the Syk kinase signaling pathway, the PI3K/Akt pathway, and the Rac/Cdc42 GTPase signaling cascades. Collectively, these pathways orchestrate the processes of phagocyte recognition, engulfment, and destruction of tumor cells [70,76,77,78]. In human ovarian cancer, antibody-secreting cells generate tumor-reactive IgG antibodies targeting MMP14, demonstrating in vitro ADCP induction and tumor growth inhibition [74]. In colorectal tumors, IgG antibodies facilitate macrophage-mediated tumor phagocytosis, improving survival rates in colorectal cancer patients [79]. The formation of antigen–antibody complexes initiates the classical complement cascade, leading to the formation of the membrane attack complex (MAC) and subsequent CDC. In a murine model of breast cancer, complement activation was shown to augment immune function and T cell activation [80]. TIL-B-derived antibodies have direct anti-tumor effects, targeting tumor antigens and contributing to tumor cell killing (Figure 2). In the setting of lung carcinomas, IgG antibodies secreted by intra-tumoral plasma cells serve to undermine tumor resilience. Specifically, they accomplish this by being internalized through a mechanism involving AP-2 adaptor proteins embedded in the tumor cell membrane. Subsequent to their intracellular transit, these antibodies encounter TRIM21, triggering a cascade of events that culminate in the ubiquitination and proteasomal degradation of RhoC—a small GTPase implicated in cytoskeletal dynamics and cell motility. By orchestrating the targeted removal of RhoC, IgG effectively restrains tumor progression, showcasing a novel facet of adaptive immunity’s impact on malignancy suppression [81]. In murine models of ovarian carcinoma, IgA derived from TIL-B cells exerts anti-neoplastic effects through transcytosis. This process involves the inhibition of the RAS pathway and sensitizes tumor cells to T cell-mediated cytotoxicity, thereby delaying tumor progression [60].

B cells can directly target and kill tumor cells, as well as enhance T cell responses. Existing research has substantiated that due to B cells’ capability to cross-present antigens, TIL-B can relay tumor-associated antigens to T cells. The underlying mechanism encompasses B cells presenting antigens via MHC class I and II, delivering co-stimulatory signals through CD80/CD86 interacting with CD28 on T cells, secreting cytokines such as CCL22 and CCL17, all of which collectively bolster T cell responses. This includes the secretion of Interferon-gamma (IFN-γ), GZMB, and other factors, ultimately leading to tumor cell lysis [82,83,84]. In vitro experiments demonstrated that cancer antigen-specific CD21lowCD86+ B cells from colorectal cancer patients can stimulate CD3+ T cells to secrete IFN-γ [52]. TIL-Bs in lung cancer activate IFN-γ responses in CD4+ T cells [51]. In HPV-positive oropharyngeal squamous cell carcinoma, an increase in TIL-Bs is closely associated with enhanced CD8+ T cell immune function [85]. As illustrated in Figure 2, GZMB, a key effector molecule in immune cell anti-tumor activity, can be produced by B cells upon BCR recognition of tumor antigens, directly killing tumor cells [86]. The mechanism by which B cells contribute to tumor cell demise involves the generation of GZMB, which upon entry into tumor cells—either via endocytosis or other means—proceeds to cleave proteins associated with apoptosis, notably members of the caspase family, found predominantly in mitochondria and the cytoplasm. By instigating DNA damage and mitochondrial dysfunction, GZMB sets off a cascade that culminates in tumor cell death [87]. Interestingly, B cell production of GZMB does not always equate to tumor killing; studies have found that IL-21-stimulated Bregs producing GZMB often coincide with Treg activity, indicating an immunosuppressive role [88]. The multifaceted anti-tumor activities of B cells are evident, with specific mechanisms and the contributions of distinct B cell subsets to tumor control remaining active areas of research.

### 3.2. B Cells: Unexpected Drivers in Tumor Evolution

In the TME, the facilitation of tumor growth is largely credited to specific B cell subpopulations with immunosuppressive properties, commonly referred to as Breg cells. Bregs make up less than 10% of circulating B cells in a normal physiological state [89]. The pro-tumorigenic activities of Bregs are fundamentally linked to signaling pathways involving IL-10, TGF-β, and the PD-1/PD-L1 immune checkpoint (Figure 2). Secreted by Bregs, IL-10 binds to its receptor on the surface of T cells and DCs, triggering JAK1 and TYK2 activation. Subsequently, these enzymes phosphorylate and activate STAT3, which translocates to the nucleus to drive the expression of anti-inflammatory genes. Concurrently, STAT3 suppresses pro-inflammatory cytokine production and restricts T cell activation, thereby indirectly fostering tumor progression [65]. TGF-β, another key cytokine released by Bregs, engages its receptor on T cells, instigating the activation of Smad2 and Smad3. Following their phosphorylation, these proteins bind to Smad4, assembling a complex that moves into the nucleus. In this compartment, the complex controls the expression of targeted genes, promoting the development of Tregs while constraining the activation of Effector T Cells (Teffs). This dual action aids tumors in circumventing immune recognition and eradication [90]. Moreover, the PD-1/PD-L1 interaction serves as a crucial component of the immunological checkpoint system. Binding of PD-L1 on B cells to PD-1 on T cells restrains T cell receptor signaling, decreasing the activity of pathways including PI3K/Akt and RAS/MEK/ERK. Consequently, there is a reduction in T cell proliferation, a decline in cytokine secretion, and a weakening of cytotoxic functions, collectively contributing to an environment favorable for tumor proliferation [91]. In addition, B cells have been shown to exacerbate tumor progression through the secretion of IL-1β. This cytokine encourages the production of factors such as Zeb1 by tumor cells, promoting angiogenesis and fueling tumor growth [92].

The Breg subtypes vary across different TMEs, with distinct functions specific to particular tumor types. A study identified a subset of B cells capable of secreting pro-angiogenic cytokines, diverging from the conventional Breg phenotype [93]. In pancreatic cancer, however, B cells support tumor progression through the production of IL-35 rather than IL-10, with immunosuppressive effects confirmed in mouse models [94]. A newly identified B cell subset contributes to tumor immune evasion in hepatocellular carcinoma by inhibiting T cell responses through PD-1 and IL-10 [56,95]. Similarly, in hepatocellular carcinoma, B cell activation by dendritic cells via IL-10 aids in the tumor’s evasion of immune surveillance [96]. Interestingly, B cells can contribute to tumor progression through the formation of immune complexes [32,97]. Notably, during antibody responses, pro-tumorigenic effects can occasionally arise. A mouse model of breast cancer demonstrated that primary tumors induced an aberrant immune response, leading to the production of IgG antibodies targeting glycosylated membrane protein HSPA4 and ultimately promoting tumor progression. These antibodies activate downstream HSPA4-binding proteins, facilitating tumor metastasis [98]. In squamous cell carcinoma, FcγR+ mast cells and macrophages recognized antibody–antigen complexes, leading to tissue remodeling, angiogenesis, and tumor progression [99]. Tumor cells are not passive players in this process; for instance, thymoma and melanoma cells can actively stimulate B cells to produce IL-10, which subsequently suppresses CD8+ T cell or NK cell IFN-γ expression, thereby promoting tumor growth [100]. Similarly, PD-L1+ Bregs in melanoma patients can facilitate tumor metastasis by inhibiting T cell responses [101]. The contrasting roles of B cell subsets, which range from fostering tumor progression to mounting defenses against it, emphasize the intricate landscape of B cell diversity within the TME. Yet, within the intricate milieu of the TME, B cells and T cells manifest dual properties—both anti-tumorigenic and pro-tumorigenic—with mechanistic distinctions underlying these paradoxical behaviors (Table 1). A deep understanding of B cell functions will pave the way for the development of next-generation cancer immunotherapies.

## 4. B Cells in Cancer Therapy

Until now, tumor immunology research has primarily focused on the anticancer functions of T cells, driving advancements in T cell immunotherapies. This focus has unveiled complex dynamics of lymphocyte infiltration in the TME, exposing tumor-induced immune suppression mechanisms [102]. The recognized correlation between B cell activity and patient prognosis in various tumor types has underscored the potential of B cell-targeted interventions. Targeting B cells shows promise in amplifying anti-tumor immune responses and enhancing chemotherapy efficacy, thereby inhibiting tumor growth through immunosuppression mitigation. This underscores the potential of B cell-based immunotherapeutic approaches. Here, we summarize preclinical methods utilizing TIL-Bs for cancer treatment (Table 2), emphasizing the enormous potential of B cells in cancer immunotherapy.

### 4.1. Enhancing B Cell Anti-Tumor Pathways

**Figure 3:**
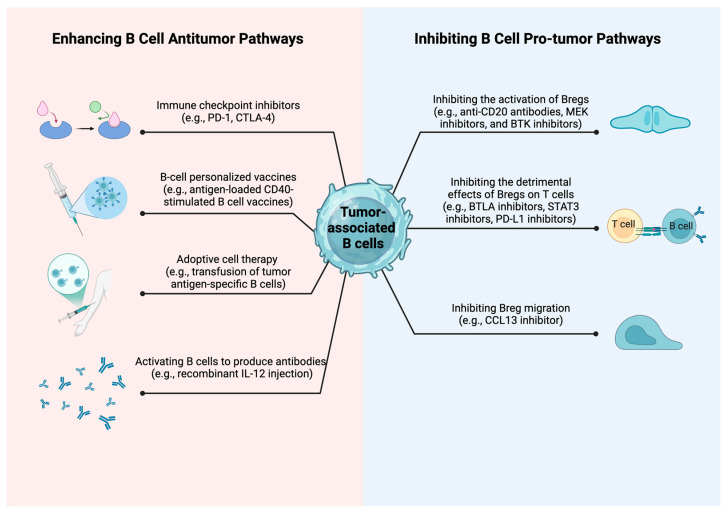
B cells in cancer therapy. In cancer treatment, strategies concerning B cells fall into two major categories: Firstly, enhancing B cell anti-tumor pathways, which comprises interrupting immune checkpoints, creating customized B cell vaccines, implementing adoptive B cell therapies, and augmenting B cells’ capability to produce antibodies; secondly, inhibiting B cell pro-tumor pathways, encompassing the suppression of Breg activation, curtailing the adverse effects of Bregs on T cells, and restraining Breg migration. Abbreviations: PD-1, programmed cell death protein 1; CTLA-4, cytotoxic T-lymphocyte-associated protein 4; IL-12, interleukin-12; MEK, mitogen-activated protein kinase; BTK, Bruton’s tyrosine kinase; BTLA, B and T lymphocyte attenuator; STAT3, signal transducer and activator of transcription 3; PD-L1, programmed cell death protein 1 ligand; CCL13, chemokine ligand 13. Created in BioRender. (accessed on 10 October 2024) https://BioRender.com/s13u901.

The remarkable anti-tumor properties of B cells have spurred the development of innovative targeted therapies that directly engage B cell function (Figure 3). B cells express inhibitory checkpoint molecules such as PD-1, PD-L1, and CTLA-4, which play critical roles in regulating immune responses. Checkpoint blockade therapy has been found to effectively activate B cells, leading to the production of tumor-reactive antibodies and enhanced B cell anti-tumor activity in a murine breast tumor model [42]. Anti-CD20-mediated B cell depletion in melanoma patients led to decreased tumor-associated inflammation and CD8 T cell counts, with plasmablast-like cells demonstrating increased PD-1 T cell activation following in vitro anti-PD-1 blockade [111]. In patients with human papillomavirus (HPV)-associated cancers, the combination of radiotherapy and PD-1 blockade potentiates B cell activity, ultimately improving outcomes for individuals suffering from HPV-related squamous cell carcinoma [112]. The use of anti-PD-1 therapy in checkpoint blockade has revealed that targeting T cells can also positively impact B cell function, leading to improved outcomes in non-small cell lung cancer patients [113]. However, the use of anti-CTLA-4 in checkpoint blockade carries the risk of adverse events, including autoimmune reactions, due to the production of autoantibodies in a subset of patients. These adverse events can limit the clinical use of anti-CTLA-4 therapy [114].

Beyond checkpoint blockade, therapeutic cancer vaccines are emerging as a promising avenue for inducing TLS and TIL-B responses. While B cell-specific personalized vaccines are currently limited, other types of cancer vaccines have shown significant potential. HPV antigen-targeting vaccines for cervical intraepithelial neoplasia have been shown to induce TLS within tumors, correlating with improved clinical outcomes [115,116]. Immunization with CD40-stimulated B cells loaded with antigens has been demonstrated to effectively activate dendritic cells and T cells, leading to a robust immune response [103]. Similarly, adoptive therapy utilizing B cells holds considerable promise. In a colon cancer model, transferring mRNA encoding carcinoembryonic antigen into B cells prior to their reinfusion effectively mediated anti-tumor immunity, consequently curtailing tumor growth [117]. In studies of synovial sarcoma, B cells, upon engulfing tumor-associated antigen DNA, demonstrated an ability to elicit T cell responses ex vivo. This finding indirectly underscores the potential of B cells in the development of tumor vaccines [118]. The impact of tumor-specific B cells on neoplasms hinges on their tumor-specific BCRs. Adoptive therapies exploiting these unique BCRs have begun implementation. In a murine breast cancer model, tumor-draining lymph node-resident B cells, after being cultured ex vivo and then reinfused, could directly eradicate tumor cells via mechanisms involving CXCR4/CXCL12 and perforin [119].

Given the antibody-producing capacity of B cells, antibody-based therapies aimed at enhancing B cell activity are also being explored. Fucoidan-rich polysaccharides from Ganoderma lucidum exert anti-tumor effects by inducing B cell-mediated IgM production, resulting in tumor cell lysis [104]. Recombinant IL-12-mediated activation of B cells and induction of antibody production in squamous cell carcinoma patients has demonstrated encouraging outcomes [120]. For ovarian cancer patients, despite the relatively poorer prognoses associated with immune checkpoint inhibitor use, an encouraging observation is that polyclonal IgA antibodies, formed through humoral immunity, bind to the ubiquitously expressed polymeric IgA receptor on ovarian cancer cells. This binding triggers transcytosis and orchestrates T cell and B cell reactions, thereby inhibiting the progression of ovarian cancer [60]. A 2017 study focusing on B cells in gastric cancer revealed that glycosaminoglycans (GAGs) serve as primary antigens recognized by gastric tumor-infiltrating B cells. Intriguingly, natural anti-sulfated GAG antibodies found in gastric cancer exhibit potent cytotoxicity against cancer cells in human lung tumors, pancreatic ductal adenocarcinoma, and colorectal cancer [121]. However, it is crucial to determine the specificity of antibodies, as some may have pro-tumor effects and be associated with autoimmunity, necessitating careful design to achieve anti-tumor effects [122].

### 4.2. Inhibiting B Cell Pro-Tumor Pathways

Given the pro-tumorigenic aspect of B cells, therapeutic strategies specifically targeting this facet are progressively emerging in cancer treatment (Figure 3). CD20-based treatments were initially explored as a means to eliminate B cells and counteract their immunosuppressive effects [123]. Research has demonstrated that B cell-deficient mice are less susceptible to squamous cell carcinoma growth, and depleting B cells with CD20 monoclonal antibodies before chemotherapy can improve the effectiveness of platinum and paclitaxel regimens [97]. Similarly, CD20-targeted therapy has demonstrated significant efficacy in suppressing tumor growth in pancreatic and squamous cell carcinomas [105]. The depletion of CD20+ B cells, primarily achieved through the binding of CD20 antibodies to B cells, disrupts the signaling cascade initiated by the BCR, affecting downstream molecules such as PI3K, BTK, and SYK. Following B cell removal, the suppression of these signaling pathways restrains tumor progression [124]. The elevated expression of B and T lymphocyte attenuator (BTLA) on B cells highlights the potential for targeting this molecule to enhance anti-tumor immunity. Clinical studies have demonstrated that combining BTLA inhibitors with chemotherapy can lead to improved outcomes [125]. Inhibiting BTLA effectively impedes the transmission of the BTLA/HVEM pathway, consequently reducing the suppression of T cells [126]. By targeting STAT3, BTK, and MEK pathways, these inhibitors can effectively enhance anti-tumor effects, providing an alternative to CD20 therapy with fewer adverse reactions [106,107,108]. Specifically, STAT3, which is highly active in Bregs, when inhibited, reduces IL-10 secretion. For MEK and BTK, their inhibition blocks the MEK/ERK and BCR downstream pathways critical for Breg activation, leading to decreased Breg activity and diminished pro-tumorigenic actions [127,128,129]. As previously mentioned, IL-10 plays a critical role in enabling Bregs to exert pro-tumoral functions. Treatment with Total glucosides of peony (TGP) decreases IL-10 production by Bregs. This effect may stem from TGP’s ability to inhibit B cell-activating factor (BAFF), contributing to its beneficial therapeutic action against hepatocellular carcinoma (HCC) [130]. Certainly, given PD-1’s inhibitory effect on T cells, combining ibrutinib with an anti-PD-L1 antibody has been found to potentiate T cell function, demonstrating promising therapeutic outcomes in breast and colorectal cancers [131]. Recent studies have demonstrated that B cell-specific deletion of IL-35 promotes plasma cell differentiation and enhances IgG and IgM production, thereby intensifying anti-tumor activity. This enhancement is attributed to the reduced influence of IL-35 on downstream STAT3-PAX5 signaling [73]. Cytokine therapies targeting B cells are being investigated as potential treatments for cancer. In pancreatic ductal adenocarcinoma, the use of CXCL13 antibodies to inhibit the migration of Bregs has demonstrated promising anti-tumor effects [109]. Similarly, in breast cancer, CXCL13 conjugated with CpG oligonucleotides to stimulate B cells and inhibit Breg cells has been found to reduce tumor metastasis [110]. Given the significant side effects associated with B cell depletion, inhibiting the pro-tumor activities of Breg cells represents a more promising approach. This strategy not only mitigates the adverse effects of B cell depletion but also specifically targets the immunosuppressive functions of Breg cells, thereby potentially enhancing the anti-tumor immune response without compromising overall immune function.

In summary, the inhibition of B cell pro-tumor pathways through various strategies, including B cell depletion, B cell checkpoint modulation, cytokine therapy, and Breg cell inhibition, offers a range of potential therapeutic avenues for cancer treatment. These approaches seek to modulate the immunosuppressive functions of B cells without compromising their anti-tumor activities, aiming to optimize the immune response within the TME.

## 5. Conclusions

The TME is characterized by a substantial presence of B cells, which, as a subset of TILs known as TIL-B, are emerging as key players in anti-tumor immunity in human cancers. B cells play a critical role in shaping the immune response within the TME, not only through antibody production but also through interactions with other immune cell types. However, TIL-B cells are a heterogeneous population, with distinct B cell subsets exhibiting varied functions in the tumor context. The disparate effects of B cell heterogeneity on tumor outcomes underscore the need for further research to elucidate the mechanisms underlying B cell subset function in cancer and to develop targeted therapies. B cells in the TME play a complex role in tumor progression, with the ability to both promote and inhibit anti-tumor immunity through their production of various factors. Acknowledging the heterogeneity of TIL-B cell subsets is imperative for advancing cancer treatment strategies. Early research often overlooked the heterogeneity of TIL-B cells due to limitations in technology. However, advancements in flow cytometry, single-cell technologies, and other methodologies have allowed for a more detailed analysis of B cell subsets within the TME. Dissecting the functions of distinct TIL-B cell subsets paves the way for a comprehensive exploration of B cell function within the TME, providing valuable insights for future research. Gaining insights into the differential characteristics of B cell subsets within the TME and the mechanisms underlying their tumor-modulating functions will provide valuable leads for identifying novel therapeutic targets and developing next-generation immunotherapies. This, in turn, holds the potential to significantly expand the therapeutic armamentarium and improve the quality of life for cancer patients. In conclusion, understanding B cell subset heterogeneity within the TME is a critical step in advancing the field of cancer immunology and developing innovative therapeutic approaches. This recognition opens up new avenues for research and therapeutic intervention, paving the way for personalized medicine.

## Figures and Tables

**Table 1:** A Brief Comparative Overview of the Dual Nature Mechanisms of B Cells and T Cells in the TME.

Mechanism Category	Anti-Tumor Mechanisms (B Cells)	Pro-Tumor Mechanisms (B Cells)	Anti-Tumor Mechanisms (T Cells)	Pro-Tumor Mechanisms (T Cells)
Direct Cytotoxicity	Directly killing tumor cells by releasing GZMB	No direct pro-tumor cytotoxicity	Directly kill tumor cells by releasing perforin and granzymes	No direct pro-tumor cytotoxicity
Antibody-mediated	Tumor killing mediated through ADCC, ADCP, and CDC.	No antibody-mediated pro-tumor mechanisms	No direct antibody-mediated mechanisms, but assist B cells in producing specific antibodies	No antibody-mediated pro-tumor mechanisms
Cytokine Secretion	Secrete cytokines (e.g., CCL17, CCL22) to enhance immune response	Secrete immunosuppressive cytokines (e.g., IL-10, TGF-β) to inhibit anti-tumor immune response	Secrete cytokines (e.g., IFN-γ) to enhance immune response	Secrete pro-tumor cytokines (e.g., IL-10) in the TME
Antigen Presentation	Provide antigen presentation function to activate T cells	No pro-tumor antigen presentation mechanisms	Directly respond by recognizing tumor antigens on antigen-presenting cells	No pro-tumor antigen presentation mechanisms

Abbreviations: GZMB, granzyme B; ADCC, antibody-dependent cell-mediated cytotoxicity; ADCP, antibody-dependent cellular phagocytosis; CDC, complement-dependent cytotoxicity; CCL17, chemokine ligand 17; CCL22, chemokine ligand 22; IL-10, interleukin 10; TGF-β, transforming growth factor-beta; IFN-γ, interferon-gamma.

**Table 2:** Commonly Used Preclinical Approaches for Directly Targeting B Cells in Cancer Therapy.

Immunotherapy	Method	Cancer Type	Outcome	References
Immune checkpoint blockade	Bioxcel BE0164 (anti-CTLA4), Bioxcel BE0146 (anti-PD1)	Breast cancer	Promoting anti-tumor response	[42]
Vaccine	Antigen-loaded CD40 B cell vaccination	Melanoma	Enhancing anti-tumor immunity	[103]
Antibody induction	L-fucose-rich polysaccharide fraction of Ganoderma lucidum	Lung cancer	Reducing tumor growth	[104]
Breg depletion	CD20-specific monoclonal antibody	Squamous cell carcinoma	Reducing tumor growth	[97]
	CD20-specific monoclonal antibody	Pancreatic ductal adenocarcinoma	Reducing tumor growth	[105]
	Lipoxin A4	Hepatocellular carcinoma	Reducing tumor growth	[106]
	MEK inhibitor	Colon cancer	Enhancing anti-tumor immunity	[107]
	Tirabrutinib	Pancreatic ductal adenocarcinoma	Enhancing anti-tumor immunity	[108]
Cytokine therapy	Anti-CXCL13 inhibitor	Pancreatic ductal adenocarcinoma	Reducing tumor growth	[109]
	CXCL13-conjugated CpG oligonucleotides	Breast cancer	Decreasing tumor metastasis	[110]

Abbreviations: Breg, B regulatory cells; MEK, mitogen-activated protein kinas.
